# Association between serum calcium levels and the risk of invasive mechanical ventilation in COPD: a retrospective cohort study

**DOI:** 10.3389/fmed.2025.1721887

**Published:** 2025-12-17

**Authors:** Min Li, Yiqing xie, Wei Lv, Denghua Ren, Qian He

**Affiliations:** 1 Department of Respiratory and Critical Care Medicine, Xishan People’s Hospital of Wuxi City, Wuxi, China; 2 Department of Respiratory and Critical Care Medicine, The Third Affiliated Hospital of Soochow University, Changzhou, China; 3 Department of Respiratory and Critical Care Medicine, Changzhou Medical Center, Changzhou First People’s Hospital, Nanjing Medical University, Changzhou, China; 4 College of Pharmacy, Anhui Medical University, Hefei, China; 5 Department of Pharmacy, Anhui Zhongke Gengjiu Hospital, Hefei, China

**Keywords:** chronic obstructive pulmonary disease, invasive mechanical ventilation, MIMIC-IV, risk, serum calcium

## Abstract

**Background:**

Disturbances in serum calcium levels have been associated with poor prognosis in various diseases. However, research regarding the relationship between serum calcium and the risk of invasive mechanical ventilation in patients with chronic obstructive pulmonary disease (COPD) remains limited. This study aims to investigate the association between serum calcium levels and the need for invasive mechanical ventilation in COPD patients.

**Methods:**

We utilized data from patients diagnosed with COPD in the MIMIC-IV database. The cohort was stratified into two groups: patients who required invasive mechanical ventilation in the intensive care unit (ICU) and those who did not. Various analytical methods, including univariate and multivariate logistic regression models, were employed to examine the association. Additionally, restricted cubic splines (RCS) were used to illustrate the relationship.

**Results:**

Our study included a total of 2876 COPD patients, with 1022 in the invasive mechanical ventilation (IMV) group and 1854 in the non-mechanical ventilation (non-MV) group. Serum calcium levels in the IMV group were significantly lower than those in the non-MV group. After adjusting for confounding variables, reduced serum calcium levels were associated with an increased risk of invasive mechanical ventilation in COPD patients (odds ratio [OR] = 1.33, 95% confidence interval [CI]: 1.05–1.68). The RCS plot demonstrated that when serum calcium levels fell below 8.45 mg/dL, the risk of invasive mechanical ventilation increased with declining calcium concentrations. In subgroup analysis, COPD patients with cerebrovascular disease showed an elevated risk of requiring invasive mechanical ventilation.

**Conclusion:**

Chronic obstructive pulmonary disease patients requiring invasive mechanical ventilation exhibit significantly lower serum calcium levels compared to those not undergoing mechanical ventilation. Hypocalcemia is associated with an increased risk of invasive mechanical ventilation in COPD patients, particularly among individuals with concomitant cerebrovascular diseases.

## Background

Chronic obstructive pulmonary disease (COPD) is a progressive lung condition characterized by increasing breathlessness and airflow limitation. Patients with severe COPD often require intensive care unit admission due to acute exacerbations or respiratory failure, potentially necessitating invasive mechanical ventilation (IMV) (1). IMV use is associated with complications such as ventilator-associated pneumonia, prolonged sedation, and increased healthcare costs (2–4). Identifying modifiable risk factors for IMV in COPD could inform preventive strategies and improve patient outcomes (5).

Calcium (Ca) is the most abundant mineral in the human body and plays a vital role in various physiological processes, including nerve transmission, muscle contraction, blood coagulation, and immune function. Approximately 98% of the body’s calcium is stored in the bones, while the remaining 2% circulates in the bloodstream. Of the total serum calcium, approximately half exists in the ionized form (iCa, the biologically active fraction), with the remainder bound to proteins (mainly albumin) or complexed in a diffusible form. Due to its measurement convenience and widespread availability in clinical practice, total calcium is commonly used as a surrogate marker for assessing calcium status (6).

Previous studies have established that decreased serum calcium levels serve as independent predictors of mortality in patients with acute ischemic stroke (7), heart failure (8), acute kidney injury (AKI) (9) and cardiogenic shock (6). Furthermore, hypocalcemia has been linked to poor prognosis in patients with sepsis (10). Patients with severe hypocalcemia that is not promptly corrected exhibit significantly higher mortality rates. Notably, hypocalcemia has been identified as an independent risk factor for adverse outcomes, including the need for IMV, in COVID-19 patients (11). However, the temporal relationship and specific association between serum calcium levels and the risk of requiring IMV in patients with COPD remain inadequately explored. This study aimed to investigate the association between serum calcium levels, measured at ICU admission, and the subsequent risk of invasive mechanical ventilation in patients with COPD.

## Materials and methods

### Data source

This study utilized data from the Medical Information Mart for Intensive Care IV (MIMIC-IV, version 2.0) database. Patients with a diagnosis of chronic obstructive pulmonary disease (COPD) were included in the analysis. The research adhered to ethical guidelines for the use of de-identified clinical data. The lead author completed the National Institutes of Health (NIH) online course “Protecting Human Research Participants” (Certification Number: 49872601). As the database contains fully anonymized patient information, the requirement for informed consent was waived. The study protocol was approved by the Institutional Review Boards of both the Massachusetts Institute of Technology (MIT) and Beth Israel Deaconess Medical Center, which oversee the MIMIC-IV database.

### Study population

Patients data were extracted from the MIMIC-IV database for the period spanning 2008–2019. The inclusion criteria comprised: (1) adult patients (age ≥ 18 years) admitted to the ICU; (2) a documented diagnosis of COPD, defined by International Classification of Diseases, Tenth Revision (ICD-10) codes (J44, J440, J441, J449). Exclusion criteria were: (1) patients with secondary or higher ICU admissions; (2) hospital or ICU length of stay less than 24 h; (3) absence of serum calcium measurements during the ICU stay; (4) patients receiving only non-invasive ventilation during their ICU stay. After applying these criteria, a total of 2867 COPD patients were included in the final cohort. Based on the administration of invasive mechanical ventilation during the ICU stay, patients were categorized into two groups: the invasive mechanical ventilation (IMV) group and the non-mechanical ventilation (non-MV) group.

### Data extraction and outcomes

Data extraction was conducted using Structured Query Language (SQL) within Navicat Premium 15.0 software. The following variables were extracted for analysis: (1) baseline demographic variables: age, sex and weight; (2) Vital signs (recorded within the first 24 h of ICU admission): heart rate (HR), mean arterial pressure (MAP), peripheral oxygen saturation (SpO_2_), temperature, and respiratory rate; (3) laboratory parameters (from the first available record after ICU admission): red blood cell (RBC), hemoglobin, hematocrit, platelet, white blood cell (WBC), anion gap, bicarbonate, chloride, creatinine, blood urea nitrogen (BUN), calcium (total calcium, measured in mg/dL), glucose, International Normalized Ratio (INR), prothrombin time (PT), sodium and potassium; (4) comorbidities: myocardial infarct, congestive heart failure (CHF), cerebrovascular disease, liver disease, renal failure, sepsis and diabetes; (5) severity of illness scores: Acute Physiology Score III (APS III) and Simplified Acute Physiology Score II (SAPSII score). Additionally, data on renal replacement therapy (RRT) and vasopressor use during the ICU stay were collected.

### Statistical analysis

The handling of missing data was performed as follows. First, we assessed the proportion of missing data for all candidate variables. Variables with more than 10% missing values were excluded from the multivariate model to avoid the potential bias and instability associated with high rates of imputation. The excluded variables for this reason were: globulin, total protein, albumin, neutrophils, eosinophils, lymphocytes, monocytes, and fibrinogen. For the remaining variables included in the final model (e.g., hematocrit, hemoglobin, platelets, WBC, BUN, glucose, INR, PT, respiratory rate, temperature, weight), the proportion of missing data was less than 10%. For these, we used mean imputation for continuous variables to retain the full sample size for analysis and to avoid the potential selection bias associated with a complete-case analysis. The normality of continuous variables was assessed using the Shapiro-Wilk test, which indicated a non-normal distribution; therefore, all continuous variables are expressed as median with interquartile range (IQR). Group comparisons for continuous variables were performed using the Mann-Whitney U test, while categorical variables are presented as frequencies and percentages and were compared using the chi-square test or Fisher’s exact test, as appropriate. Based on established clinical reference ranges (12), patients were categorized into three groups according to serum total calcium levels: normal calcium group (8.8–10.4 mg/dL), hypocalcemia group (<8.80 mg/dL), and hypercalcemia group (>10.4 mg/dL). The relationship between serum calcium categories and the risk of IMV was analyzed using both univariate and multivariate logistic regression models. Results are presented as odds ratios (ORs) with 95% confidence intervals (CIs). Three models were constructed: Model 1 was unadjusted; Model 2 was adjusted for age and sex; Model 3 was further adjusted for age, sex, weight, heart rate, MAP, respiratory rate, temperature, SpO_2_, RBC, hemoglobin, hematocrit, platelet, WBC, anion gap, bicarbonate, chloride, creatinine, glucose, INR, PT, sodium, potassium, myocardial infarct, CHF, cerebrovascular disease, liver disease, renal failure, sepsis, diabetes, APS III, SAPS II, vasopressor and RRT. Restricted cubic spline (RCS) plots were used to illustrated the potential non-linear relationship between serum calcium levels and the risk of IMV. All statistical analyses were performed using R software (version 4.2.3) and IBM SPSS Statistics (version 23.0). A significance level of *p* < 0.05 was considered statistically significant.

## Results

### Baseline characteristics

**FIGURE 1:**
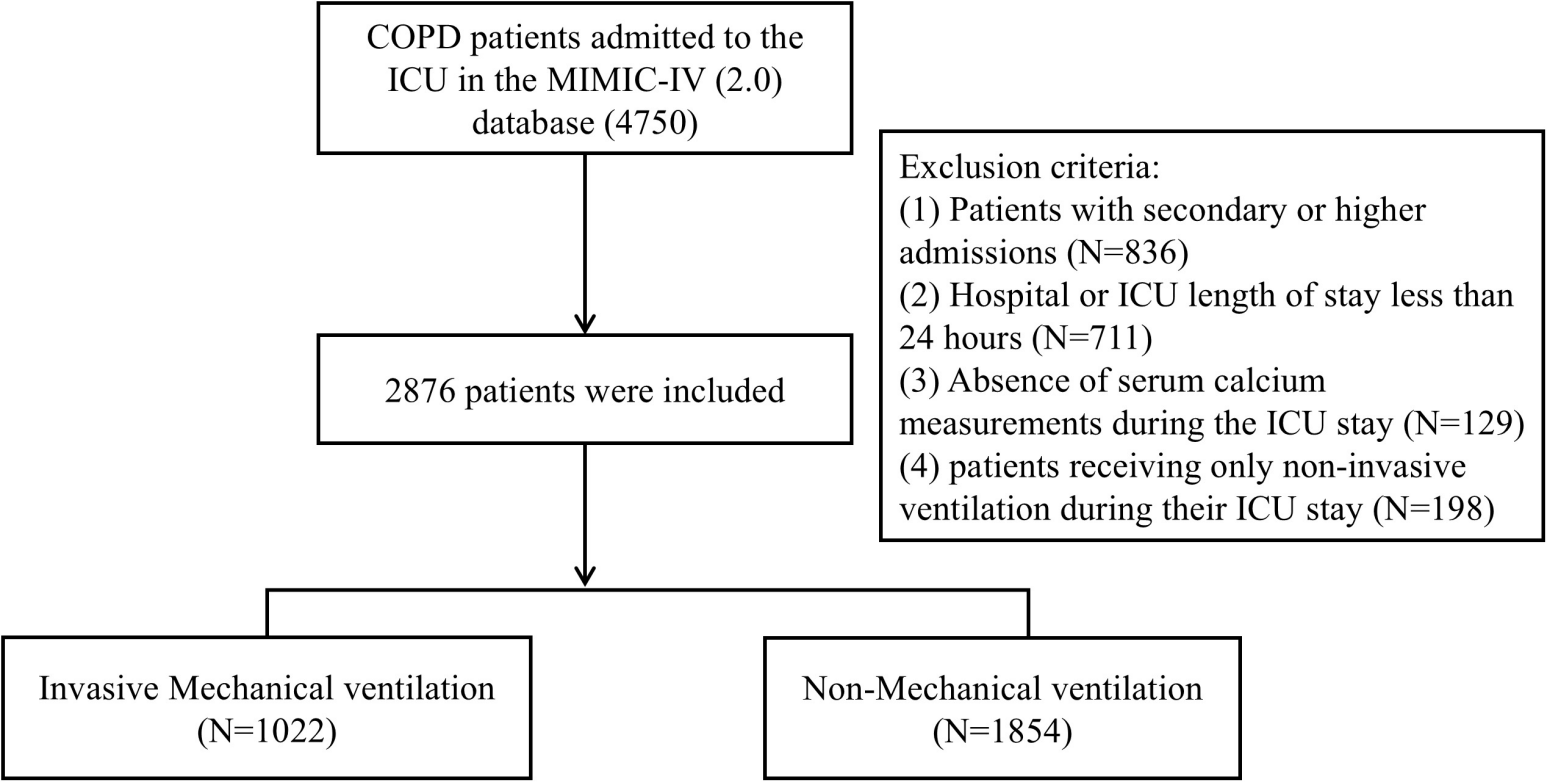
The flow chart of the included population.

This study included a total of 2876 patients with COPD (Figure 1). Participants were divided into two groups according to invasive mechanical ventilation status during the ICU stay: the IMV group, consisting of 1022 cases, and the non-MV group, comprising 1854 cases.

Table 1 presents detailed demographic characteristics, vital signs, laboratory indicators, and baseline comorbidities for both groups. Compared to the non-MV group, patients in the IMV group showed significantly lower HR, MAP, platelet counts, and bicarbonate levels (all *P* < 0.05). Conversely, the IMV group had significantly higher WBC, BUN, chloride, glucose, creatinine, potassium, SAPS II scores and APS III scores. Additionally, the median serum calcium level in the IMV group was significantly lower than that in the non-MV group (8.3 vs. 8.55 mg/dL, *P* < 0.001). The distribution of patients across calcium categories also differed significantly between the two groups (*P* < 0.001).

**TABLE 1:** Baseline characteristics of COPD patients.

Characteristic	Non-MV	IMV	*P*
*N*	1854	1022	
Age, years	72.23 (63.92, 80.79)	71.37 (63.87, 78.41)	0.008
**Sex, *n***			
Female	907	450	0.012
Male	947	572	
Weight, kg	76.72 (63, 92.4)	81.85 (67, 98.75)	<0.001
**Vital signs**			
HR, beats/min	84.52 (74.24, 96.92)	81.33 (73.18, 93.46)	<0.001
MAP, mmHg	77.6 (71, 86.04)	74.53 (70.44, 80.43)	<0.001
Respiratory rate, breaths/min	19.52 (17.19, 22.07)	19.40 (17.25, 21.93)	0.499
Temperature	36.79 (36.64, 36.95)	36.92 (36.7, 37.19)	<0.001
SpO_2_, %	95.76 (94.23, 97.08)	97.31 (95.95, 98.64)	<0.001
**Laboratory parameters**			
RBC, g/L	3.67 (3.19, 4.15)	3.66 (3.22, 4.11)	0.324
Hemoglobin, g/dl	10.2 (8.6, 11.8)	10.00 (8.6, 11.65)	0.303
Hematocrit, %	32 (27.29, 36.8)	31.42 (27.31, 36.24)	0.216
Platelet, 10^9^/L	198 (148, 263)	178 (134.5, 239)	<0.001
WBC count, 10^9^/L	10.8 (7.94, 14.55)	12.60 (9.21, 16.59)	<0.001
Anion gap, mEq/L	14.5 (12.5, 17)	14.5 (12, 17)	0.056
Bicarbonate, mg/dl	24 (21, 27)	23.00 (20.5, 25.5)	<0.001
BUN, mg/dL	21.5 (15, 36.5)	23.25 (15.5, 38.5)	0.008
Chloride, mmol/L	101 (97, 104.5)	102.5 (98, 106)	<0.001
Creatinine, mg/dl	1.0 (0.75, 1.6)	1.15 (0.8, 1.84)	<0.001
Glucose, mg/dl	130 (108, 162)	137 (113, 170.5)	<0.001
Sodium, mg/dl	138.5 (135.5, 141)	138.5 (136, 141)	0.032
Potassium, mg/dl	4.25 (3.9, 4.7)	4.45 (4.05, 4.9)	<0.001
INR	1.29 (1.1, 1.49)	1.30 (1.15, 1.49)	0.280
PT	13.8 (12.1, 16.21)	14.00 (12.4, 16.21)	0.240
**Severity of illness**			
APS III score	45 (34, 57)	54 (38, 74)	<0.001
SAPS II score	35 (28, 42)	44 (36, 52)	<0.001
**Comorbidities, *n***			
Myocardial infarct	435	309	<0.001
Congestive heart failure	924	496	0.503
Cerebrovascular disease	301	155	0.453
Liver disease	189	90	0.229
Diabetes	666	408	0.034
Renal disease	536	331	0.052
Sepsis	871	744	<0.001
RRT	86	63	0.077
Vasopressor use	86	207	<0.001
**Calcium status**			
Calcium, mg/dL	8.55 (8.1, 8.95)	8.3 (7.85, 8.75)	<0.001
**Calcium category, *n***			
Normal (8.8–10.4 mg/dL)	649	242	<0.001
Hypocalcemia (<8.80 mg/dL)	1187	768	
Hypercalcemia (>10.4 mg/dL)	18	12	

### Association between serum calcium levels and the risk of invasive mechanical ventilation in COPD patients

The results of the logistic regression analysis are presented in Table 2. In the unadjusted model, hypocalcemia was significantly associated with an increased risk of invasive mechanical ventilation compared to normal calcium levels (OR = 1.74, 95% CI: 1.46–2.06). In Model 2, which was adjusted for age and sex, the association between hypocalcemia and invasive mechanical ventilation risk remained significant. Model 3, which included comprehensive adjustments for demographic characteristics, vital signs, laboratory parameters, comorbidities, illness severity scores, and treatment measures, demonstrated consistent results (OR = 1.33, 95% CI: 1.05–1.68), with a significant trend across calcium categories (P for trend = 0.017).

**TABLE 2:** Logistic regression analysis of the association between serum calcium levels and the risk of invasive mechanical ventilation.

Variables	Model 1		Model 2		Model 3	
Calcium, mg/dL	OR (95% CI)	*P*-value	OR (95% CI)	*P*-value	OR (95% CI)	*P*-value
8.8–10.4	Reference		Reference		Reference	
<8.80	1.74 (1.46, 2.06)	**<0.001**	1.71 (1.44, 2.04)	**<0.001**	1.33 (1.05, 1.68)	**0.017**
>10.4	1.79 (0.85, 3.77)	0.126	1.87 (0.89, 3.95)	0.114	0.45 (0.16, 1.24)	0.122

Bold values indicate statistical significance (*P* < 0.05). Model 1: unadjusted. Model 2: adjusted for age and sex. Model 3: adjusted for age, sex, weight, vital signs (heart rate, MAP, respiratory rate, temperature, SpO_2_), laboratory parameters (RBC, hemoglobin, hematocrit, platelet, WBC, anion gap, bicarbonate, chloride, creatinine, glucose, INR, PT, sodium, potassium), comorbidities (myocardial infarction, CHF, cerebrovascular disease, liver disease, renal failure, sepsis, diabetes), illness severity scores (APS III, SAPS II), and treatments (vasopressor use, RRT).

**FIGURE 2:**
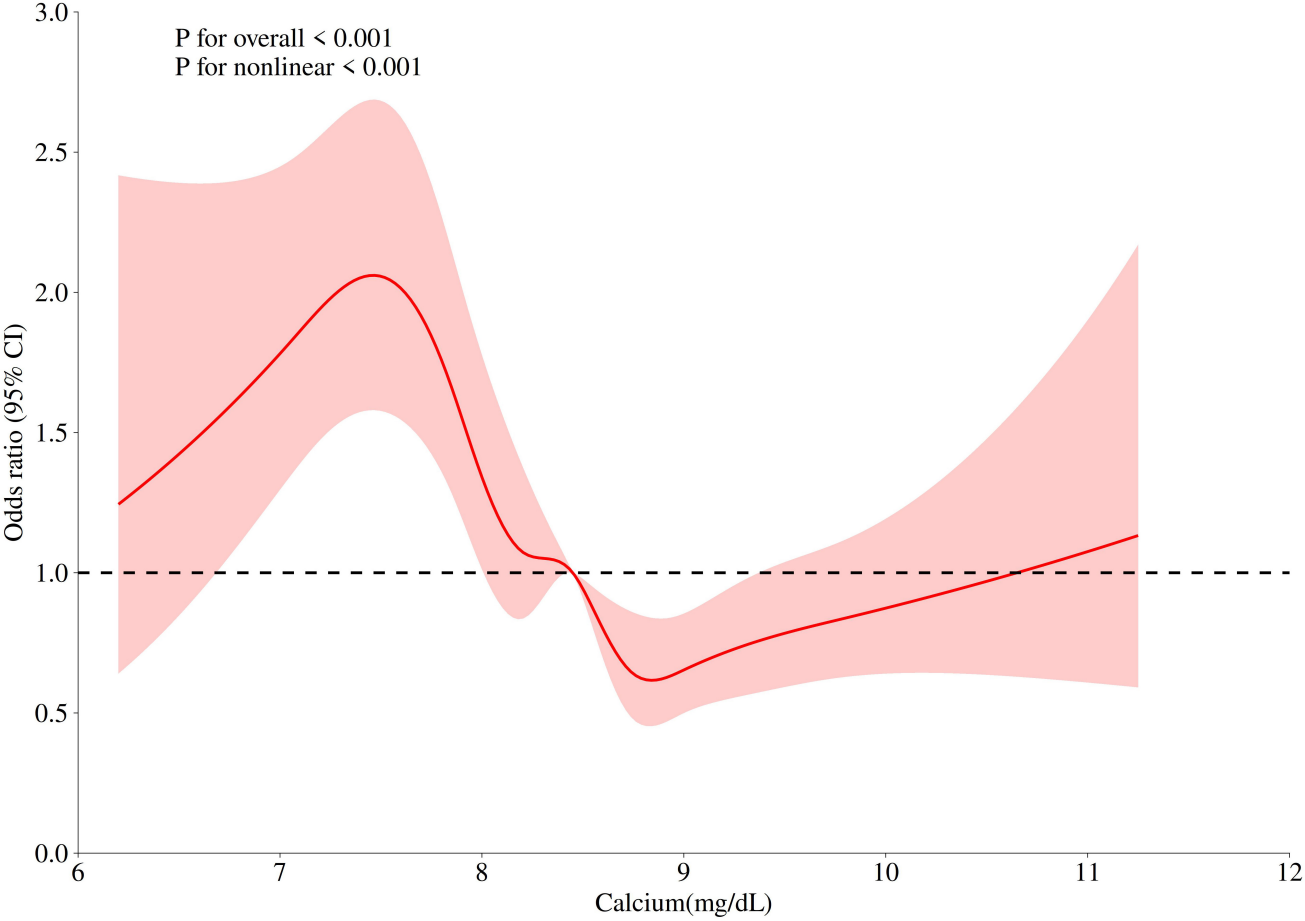
Non-linear relationship between serum calcium levels and invasive mechanical ventilation risk in COPD patients.

A significant non-linear association was observed between serum calcium levels as a continuous variable and the risk of invasive mechanical ventilation (*P* < 0.001). Specifically, the restricted cubic spline analysis revealed that when serum calcium levels decreased below 8.45 mg/dL, the risk of IMV progressively increased with declining calcium concentrations (Figure 2).

### Subgroup analysis

We performed subgroup analysis to evaluate the association between serum calcium levels and IMV risk across prespecified patient strata, including age, sex, congestive heart failure, myocardial infarction, cerebrovascular disease, renal failure, liver disease sepsis and diabetes. A significant interaction was observed for cerebrovascular disease (P for interaction = 0.039), indicating that the association between hypocalcemia and IMV risk was particularly pronounced in patients with this comorbidity. No other significant interactions were detected (Table 3).

**TABLE 3:** Subgroup analysis of the association between serum calcium levels and the risk of invasive mechanical ventilation in COPD patients.

Subgroup	*N*	OR (95% CI)	*P*-value	P-interaction
**Sex**				
Male	1519			0.398
8.8–10.4		Ref		
<8.80		1.79 (1.3, 2.49)	<0.001	
>10.4		0.11 (0.19, 0.6)	0.011	
Female	1357			
8.8–10.4		Ref		
<8.80		1.12 (0.81, 1.54)	0.507	
>10.4		1.32 (0.37, 4.78)	0.670	
**Age**				
≥65	2078			0.347
8.8–10.4		Ref		
<8.80		1.53 (1.17, 2.01)	0.002	
>10.4		0.61 (0.21, 1.73)	0.348	
<65	798			
8.8–10.4		Ref		
<8.80		1.04 (0.67, 1.62)	0.868	
>10.4		0.56 (0.03, 12.13)	0.712	
**Myocardial infarct**				
No	2132			0.079
8.8–10.4		Ref		
<8.80		1.31 (1.01, 1.72)	0.041	
>10.4		0.91 (0.29, 2.8)	0.866	
Yes	744			
8.8–10.4		Ref		
<8.80		1.63 (1.05, 2.53)	0.028	
>10.4		0.09 (0.01, 0.68)	0.019	
**Congestive heart failure**				
No	1456			0.692
8.8–10.4		Ref		
<8.80		1.26 (0.91, 1.76)	0.176	
>10.4		0.23 (0.04, 1.51)	0.125	
Yes	1420			
8.8–10.4		Ref		
<8.80		1.56 (1.14, 2.13)	0.006	
>10.4		0.76 (0.22, 2.6)	0.658	
**Cerebrovascular disease**				
No	2420			**0.039**
8.8–10.4		Ref		
<8.80		1.37 (1.07, 1.75)	0.215	
>10.4		0.78 (0.25, 2.4)	0.663	
Yes	456			
8.8–10.4		Ref		
<8.80		1.77 (0.95, 3.29)	0.041	
>10.4		0.08 (0.04, 1.73)	0.107	
**Renal failure**				
No	2009			0.092
8.8–10.4		Ref		
<8.80		1.62 (1.22, 2.15)	<0.001	
>10.4		0.21 (0.03, 1.3)	0.093	
Yes	867			
8.8–10.4		Ref		
<8.80		1.15 (0.78, 1.69)	0.496	
>10.4		0.79 (0.21, 2.93)	0.718	
**Liver disease**				
No				0.068
8.8–10.4	2597	Ref		
<8.80		1.47 (1.16, 1.86)	0.002	
>10.4		0.46 (0.16, 1.28)	0.137	
Yes	279			
8.8–10.4		Ref		
<8.80		0.91 (0.39, 2.11)	0.827	
>10.4		–	–	
**Diabetes**				
No	1802			0.529
8.8–10.4		Ref		
<8.80		1.69 (1.26, 2.27)	<0.001	
>10.4		0.49 (0.13, 1.82)	0.286	
Yes	1074			
8.8–10.4		Ref		
<8.80		1.22 (0.85, 1.75)	0.286	
>10.4		0.47 (0.1, 2.27)	0.345	
**Sepsis**				
No	1261			0.360
8.8–10.4		Ref		
<8.80		0.86 (0.57, 1.31)	0.485	
>10.4		0.42 (0.04, 4.32)	0.465	
Yes	1615			
8.8–10.4		Ref		
<8.80		1.52 (1.14, 2.02)	0.004	
>10.4		0.54 (0.17, 1.72)	0.300	

Bold value indicates statistical significance (*P* < 0.05).

Confounders were consistent with the model 3 in Table 2.

## Discussion

In this retrospective cohort study, we assessed the relationship between serum calcium levels and the risk of invasive mechanical ventilation in patients with COPD. Our analysis identified an inverse correlation between serum calcium levels and the risk of IMV in this population. Subgroup analysis revealed that in patients with COPD and cerebrovascular disease, hypocalcemia was strongly associated with an increased risk of IMV compared to those with normal calcium levels. These findings may provide valuable insights for identifying COPD patients at high risk for poor prognosis.

Serum calcium plays a crucial role in various physiological processes in the human body (8, 12). Including neurotransmitters release, which affects communication between nerve cells, and its involvement in blood coagulation. Additionally, calcium helps cell membrane stability and is essential for muscle contraction (13). Previous studies have shown that serum calcium levels in COPD patients are often low, which may be related to factors such as chronic inflammation, malnutrition, vitamin D deficiency, impaired renal function, and long-term use of medications like corticosteroids (14, 15). One study comparing serum calcium levels in stable COPD and acute exacerbation of COPD (AECOPD) found that calcium levels were lower in AECOPD patients, with 56% exhibiting hypocalcemia (16). In our study, 66.5% of patients had hypocalcemia, with the higher proportion likely due to the inclusion of ICU patients, who generally have more severe conditions. Based on the severity of exacerbations, hospitals manage these cases with respiratory support and medication, including bronchodilators, corticosteroids, and antibiotics. The pharmacological use of corticosteroids can inhibit intestinal calcium absorption and stimulate tubular calcium excretion, leading to a significant negative calcium balance (17). On the other hand, severe COPD exacerbations can result in hypoxia, respiratory acidosis, and metabolic abnormalities, all of which can disrupt serum electrolyte balance.

The potential mechanisms linking hypocalcemia to an increased risk of invasive mechanical ventilation can be explained through its detrimental effects on both respiratory muscle and cardiac function. Calcium ions are critical for neuromuscular excitation-contraction coupling (18, 19). Hypocalcemia lowers the threshold for depolarization of nerve and muscle fibers, leading to neuromuscular instability. This can manifest as diaphragmatic weakness and fatigue, impairing the patient’s ability to maintain adequate ventilation (20, 21). Furthermore, severe hypocalcemia can directly precipitate laryngospasm and bronchospasm, which can acutely precipitate respiratory failure and the need for ventilatory support (22). Beyond its direct impact on respiratory muscles, hypocalcemia adversely affects cardiac performance, which can secondarily worsen respiratory status. Ionized calcium is essential for myocardial contractility. Low serum ionized calcium levels are associated with decreased left ventricular contractility and prolonged QTc interval, potentially leading to acute heart failure and pulmonary edema (21, 23). This cardiovascular compromise can exacerbate the existing ventilation-perfusion mismatch in critically ill COPD patients, pushing them toward respiratory failure. Finally, the relationship between hypocalcemia and respiratory function may be bidirectional. Acute respiratory failure, often accompanied by alkalosis, can further reduce physiologically active ionized calcium levels, creating a vicious cycle that promotes diaphragmatic fatigue and weakness, on top of the already increased load on the respiratory muscles from the underlying COPD itself (24). A study involving 25,706 patients with acute respiratory failure (ARF) found a statistically significant negative correlation between serum ionized calcium levels and the risk of requiring invasive mechanical ventilation. Specifically, when the serum calcium level at admission was below 4.8 mg/dL, low serum calcium was progressively associated with a higher risk of hospitalization for ARF (24). Additionally, a study on COVID-19 revealed that 44.8% of hospitalized individuals had low serum calcium levels, and 64.6% of intubated patients had hypocalcemia. Hypocalcemia was positively correlated with intubation/mechanical ventilation (25). In our study, we also found that in COPD patients, hypocalcemia was negatively correlated with the risk of invasive mechanical ventilation. The RCS curve demonstrated that when serum calcium levels fell below 8.5 mg/dL, the risk of invasive mechanical ventilation increased as serum calcium concentrations decreased. Similarly, in a study on patients with spontaneous subarachnoid hemorrhage, the rate of invasive mechanical ventilation was significantly higher in the hypocalcemia group compared to the normal calcium group (38% vs. 48.8%) (26). Our subgroup analysis also revealed that hypocalcemic patients with coexisting cerebrovascular diseases had an even higher risk of requiring invasive mechanical ventilation.

This study evaluates the relationship between serum calcium levels and the risk of invasive mechanical ventilation in patients with COPD. As a routinely monitored indicator in COPD patients in the ICU, serum calcium and could assist in the early assessment of the risk for invasive mechanical ventilation. This may enable timely preventive measures for high-risk COPD populations. However, Our study also has certain limitations. First, it is a retrospective analysis from a single database, and residual confounding cannot be ruled out. Most importantly, we relied on total serum calcium measurements without adjustment for albumin levels due to extensive missing data for albumin in our cohort. This is a significant limitation because hypoalbuminemia, common in critical illness, can lower total calcium without a true reduction in ionized calcium, potentially confounding our results. Secondly, while we adjusted for potential confounding factors to mitigate the inherent bias risks of retrospective cohort studies, residual bias may still exist. Furthermore, our study does not provide conclusive evidence on whether early calcium supplementation in COPD patients can reduce the need for invasive mechanical ventilation. Future, more rigorously designed studies will be needed to validate the findings of this research.

## Conclusion

This study evaluated the relationship between serum calcium levels and the risk of invasive mechanical ventilation in COPD patients. We found that hypocalcemia is associated with the risk of requiring invasive mechanical ventilation, especially in patients with cerebrovascular diseases, where the association is even more pronounced.

## Data Availability

Publicly available datasets were analyzed in this study. This data can be found here: https://physionet.org/content/mimiciv.
